# The Future Exploring of Gut Microbiome-Immunity Interactions: From In Vivo/Vitro Models to In Silico Innovations

**DOI:** 10.3390/microorganisms12091828

**Published:** 2024-09-04

**Authors:** Sara Bertorello, Francesco Cei, Dorian Fink, Elena Niccolai, Amedeo Amedei

**Affiliations:** 1 Department of Experimental and Clinical Medicine, University of Florence, 50139 Florence, Italy; sara.bertorello@unifi.it (S.B.); f.cei1@student.unisi.it (F.C.); dorian.fink@edu.unifi.it (D.F.); amedeo.amedei@unifi.it (A.A.); 2 Laboratorio Congiunto MIA-LAB (Microbiome-Immunity Axis Research for a Circular Health), University of Florence, 50134 Florence, Italy

**Keywords:** microbiome, immunity, inflammation, in vivo models, in vitro models, in silico models

## Abstract

Investigating the complex interactions between microbiota and immunity is crucial for a fruitful understanding progress of human health and disease. This review assesses animal models, next-generation in vitro models, and in silico approaches that are used to decipher the microbiome-immunity axis, evaluating their strengths and limitations. While animal models provide a comprehensive biological context, they also raise ethical and practical concerns. Conversely, modern in vitro models reduce animal involvement but require specific costs and materials. When considering the environmental impact of these models, in silico approaches emerge as promising for resource reduction, but they require robust experimental validation and ongoing refinement. Their potential is significant, paving the way for a more sustainable and ethical future in microbiome-immunity research.

## 1. Introduction

The gut microbiota (GM), a complex community of microorganisms (bacteria, archaea, fungi and protozoa) that lives in symbiosis with the organism in the gastrointestinal tract, plays a crucial role in interacting with the host’s immune system (IS). This bidirectional interaction strongly influences the host’s physiology, contributing to homeostasis and modulating both innate and adaptive immune responses [1].

The GM modulates the immune response through various mechanisms, including:

- production of metabolites such as short-chain fatty acids (SCFAs), bile acids (BAs), and tryptophan metabolites, which influence the activity of immune cells by promoting the production of regulatory T cells (Tregs) and effector T cells involved in the maintenance of immune tolerance and the prevention of excessive inflammatory responses [2,3,4];
- interaction with intestinal epithelial cells, stimulating the synthesis of antimicrobial molecules such as defensins, which help maintain the integrity of the intestinal barrier and prevent the invasion of pathogenic microbes [5];
- synthesis of lipopolysaccharides and peptidoglycans that activate Toll-like receptors (TLRs) expressed by innate immune cells, modulating cytokine production and influencing inflammatory responses [6];
- physical and functional maturation of the IS in the early years of life [7].

Alterations in the normal GM composition, known as dysbiosis, have been associated with a wide range of pathologies, including inflammatory bowel diseases (IBD), obesity, diabetes, cardiovascular diseases, neuropsychiatric and neurodegenerative disorders. Understanding how the microbiota interacts with the IS is therefore fundamental to developing new preventive, diagnostic and therapeutic strategies [8]. 

In recent years, advances in molecular biology, genomics, bioinformatics analysis and high-throughput sequencing techniques have improved our understanding of the microbiota-immune axis (MIA) and how GM is involved in regulating host’s health and various diseases [9]. This review examines animal models, next-generation in vitro models, and in silico approaches, evaluating their strengths and limitations to better understand the GM impact on host immunity and so on health and human diseases.

## 2. In Vivo Models for Studying the Microbiota-Immune System Axis

In vivo, studies of the MIA play a crucial role in understanding the GM influence on the host’s health. By utilizing experimental models, these studies allow for the manipulation of specific microbial compositions to examine how they affect immune responses and the overall well-being of the organism. These approaches not only reveal the fine mechanisms of microbiota-immune interactions but are also essential for developing innovative therapies and treatments aimed at modulating the microbiota and, in doing so, improving human health. Table 1 provides a comprehensive overview of the various in vivo models used to study these interactions. 

### 2.1. Vertebrates

Rodents

Rodent models, especially mice, have become extensively utilized in clinical research [10] and are a primary choice for investigating the gut-IS interaction. Given current ethical considerations, rodent models offer a convenient way to obtain numerous samples from various gastrointestinal (GI) sites while providing a wide range of genotypic backgrounds [11]. Primarily, the focus was on germ-free (GF) mice models, which yielded foundational insights into early host-microbe interactions [12]. However, there is a growing use of mice to explore dietary impacts, disease progression and the effects of microbial therapies. Rodent models yield an information wealth due to their anatomical, histological and physiological similarities with the human GI tract. Nevertheless, it is essential to acknowledge the differences, such as the circadian rhythm and lifestyle [13,14], as well as morphological variations and dietary habits. The anatomy of different GI tract segments in rodents diverges from that in humans, and their diet is distinct since mice and rats are typically fed standardized vegetarian diets. This nutritional way significantly contrasts with the more diverse human diets [15]. In fact, mice and rats are mainly herbivores and practice coprophagy, whereas human diets vary widely based on ethnicity, geography, culture and tradition [16].

Rats

Rats are considered preferable for microbiota studies as they provide a biological system similar to mice but with a size that better supports some experimental approaches, such as colonoscopy and surgical manipulation [17]. In addition, they exhibit physiological parameters that are more closely aligned with those of humans [17]. Fritz et al. compared various animal models used to study host-microbe interactions, noting that rats offer several advantages [18]. These include the availability of numerous rat-specific disease models, genetically modified rats with fully sequenced genomes and their relative ease of maintenance. Furthermore, their rapid reproduction rate allows for the observation of multiple generations within a short time frame, as rats typically live two to three years [18]. However, the same authors highlighted a key disadvantage: the diet and living conditions of rats differ significantly from those of humans [18]. While mouse models share many advantages with rat models, the significant drawback is the pronounced differences in IS function and microbiota composition compared to humans [18]. 

Several rat strains are commonly utilized in scientific research, including Sprague–Dawley [19], Wistar [20,21], Fischer 344 [22], Lewis [23], wild-type Groningen [24,25] and BioBreeding rats [26]. The selection of a specific strain depends on the study type and the strain’s behavioral and genetic characteristics. 

In general, rat models are particularly valuable for investigating the relationship between gut dysbiosis and disease, as well as for examining the dietary and pharmaceutical impacts on GM. For instance, the dextran sodium sulfate (DSS) colitis murine model is widely used in IBD research due to its simplicity, reproducibility and controllability [27]. Additionally, rat models are used to study different autoimmune, metabolic and neurological disorders such as diabetes [28], autism spectrum disorder (ASD) [29], Parkinson’s disease [30] and depression [31]. These models are essential for understanding the differences in GM composition between rats and controls, further highlighting the significance of the gut-immunity and gut-brain axis. 

Mostly, the intestinal microbiota of rats, mice and humans are similar at the phylum level but different at the genus level [16,18]. In rats, the dominant phyla are *Firmicutes* and *Bacteroidetes* [18], which also constitute about 90% of bacterial species in the adult human gut. A study that established a catalog of microbial genes found a higher pairwise overlap between the gut metagenome of rats and humans (2.47%) compared to that between mice and humans (1.19%) [32]. Additionally, Pan et al. highlighted the rats potential for biomedical research, noting that 97% of functional pathways present in the human catalog were also found in the rat catalog [32]. 

Li et al. conducted a study characterizing the microbiota along the longitudinal axis of the rat GI tract, including fecal samples [33]. Their results documented that the microbial composition in the rat GI tract is distinct from that of other murine models, such as mice. Specifically, species richness and phylogenetic diversity increase from the upper to the lower GI segments, with the highest levels found in the colon. In contrast, mice show similar diversity levels between duodenal and large-intestinal samples [33].

When using rat models to study the human microbiome, one relevant difference to consider is that inter-individual microbiota variability is significantly higher in humans than in rats. This human variability is attributed to genetic differences, diverse diets, and varying environmental factors, whereas laboratory rats exhibit lower variability due to their genetic similarity, uniform diet, controlled environmental conditions, and coprophagy [33]. Furthermore, the GM profile in rats changes with age and can be categorized into three distinct clusters: (1) before weaning, (2) the first year of life and (3) the second year of life. These clusters share a core set of bacterial species whose relative abundance decreases with age, while alpha-diversity increases over time [34].

Mice

Mice of the species *M. musculus* are commonly employed to systematically investigate the roles of diet, pathogens and host genotype on GM diversity [35]. Although laboratory mice are typically fed vegetarian diets, which differ from the varied diets of humans, they still provide valuable insights due to their anatomical, histological, and physiological similarities to humans. However, important differences must be considered when designing experiments and evaluating results. One major difference is the relative size of the intestinal tract compared to the overall body size, along with distinct features throughout the tract. For example, mice possess a non-glandular forestomach, which is missing in humans. This forestomach, lined with keratinizing squamous mucosa, covers two-thirds of the stomach and functions primarily as a storage site for food, lacking secretory activity [36]. It is also coated with a biofilm containing various strains of *Lactobacillus* spp. [37,38]. Although *L. reuteri* and *L. johnsonii* are distributed throughout the mouse intestinal tract, evidence suggests that the forestomach is their primary habit, with cecal populations likely derived from the forestomach [39]. Comparative genomic analysis reveals that murine strains of *L. reuteri* are significantly different from those found in humans. These strains possess urease genes to cope with the low pH of the forestomach, along with various rodent-specific genes essential for their persistence in mice [40]. 

The phylogenetic composition of bacterial communities in humans and mice is similar at the phylum level, with *Bacteroidetes* and *Firmicutes* being the dominant bacterial phyla in the murine intestinal tract [41,42]. This similarity extends to many other mammals, regardless of their diet as herbivores or carnivores [41,42]. However, there are remarkable differences between the human and mouse intestinal microbiota. One key difference is that the murine intestinal tract harbors significant populations of the phylum *Deferribacteres*, which in humans is present only in trace amounts in the stomach [43]. Additionally, mice show a unique member of the *Firmicutes*, known as segmented filamentous bacteria (SFB) or “*Candidatus arthromitus*” [44], which significantly impacts the IS maturation [45,46,47] but its role in humans is not yet fully understood. 

A comparative study involving 16 human subjects and 3 commonly used mouse lines found that while their microbiota appeared similar, it was quantitatively very different [48]. Although there are 80 shared microbial gut genera between humans and mice [15], significant variations exist in the genera observed in mouse datasets. For instance, genera such as *Faecalibacterium*, *Sucinivibrio* and *Dialister* were missing in some laboratory mice [48,49] but detected in other more comprehensive studies [15]. These discrepancies can be attributed to the use of different mouse strains and providers, as well as variations in analytical methods. For example, differences in microbial composition analysis can arise from using different 16S rRNA gene-based primers, targeted variable regions and sequencing platforms [15].

Guinea Pigs

Guinea pigs (*Cavia porcellus*), native to South America [50], offer an alternative to the more commonly used rat and mouse models in experimental research on humans. Their suitability as models for studying gastrointestinal dynamics stems from the similarities between their intestinal E-cadherin and that of humans [51]. Despite this, research on guinea pigs’ GM is less extensive compared to studies involving rats and mice. Metagenomic analysis revealed that guinea pigs possess a higher proportion of *Akkermansia* spp. and methanogenic bacteria relative to human microbiota [51]. Nevertheless, *Bacteroidetes* and *Firmicutes* are the predominant microbial phyla in the guinea pig GI tract, similar to rats, mice and typical vertebrate gut microbiomes, including humans [51,52]. However, there are prominent differences at the genus level, which may be attributed to variations in GI anatomy, diet and coprophagy. Despite these differences, guinea pigs have proven valuable for studying various human diseases, especially infections, due to their similar symptoms and immune responses to those observed in humans. Given these similarities and the overall resemblance of guinea pigs to other rodent models, *Cavia porcellus* is considered a promising model for investigating the MIA and its role in human diseases [53].

Rabbit (*Oryctolagus cuniculus*)

The rabbit model (*Oryctolagus cuniculus*) has not yet replaced the murine model, which remains favored due to its lower cost, high fertility [54], reduced maintenance costs, smaller size, availability of inbred strains, ease of breeding, and the wide availability of commercial immunological reagents and numerous knockout (KO) and transgenic models. However, the rabbit model shows some aspects making it preferable in some studies compared to mice [55].

Historically, rabbits have been extensively used for studying infectious agents such as *Vibrio cholerae* [56] and many other pathogens [57,58,59]. A recent review by Esteves et al. highlights the rabbits’ advantages over rats and mice, especially their sophisticated adaptive immune system, which provides insights into human biology and yields valuable clinical and research reagents. Additionally, rabbits are excellent models for studying diseases such as syphilis and tuberculosis, which produce pathology similar to that seen in human patients [60]. The similarity between rabbit IS and rodents has been known for years, with several studies published on this model and its role in immunological contexts [61]. Additionally, rabbits offer advantages in size; they are intermediate between rodents and larger, more expensive animal models like primates. This size allows for rapid blood sampling and greater access to various cells and tissues from a single animal. Furthermore, rabbits have a longer lifespan than rodents, and their IS genes are more similar to those of humans than those of rodents [62,63]. Regarding their GM, rabbits have been characterized at various life stages and in different parts of the GI tract. Similar to humans, *Firmicutes* is the most abundant phylum, although the species within this phylum are less abundant in rabbits compared to humans [64,65,66]. Additionally, GF models, similar to those used for rodents, are now common also in this species [67].

Pigs

Domestic and miniature pigs have become standard model species in various areas of translational research. Miniature or micro pigs are more desirable and frequently used in research due to their smaller size, making them easier to handle and manipulate, and also less costly [68,69]. The pigs’ attractiveness as research models comes from their comparable size, physiology and developmental trajectories relative to humans, as well as the ability to manipulate their genome [70]. The applications of this model are numerous, including gastrointestinal physiology and immuno-ontogeny [71]. As omnivores with a GI structure similar to humans, the well-characterized fecal microbiota of young and adult domestic pigs [72] shows compositional similarities with the human microbiota [73,74]. This similarity is mainly evident in studies of diet-induced obesity, where changes in microbiota composition, such as an increase in the *Firmicutes* to *Bacteroidetes* ratio, are observed in both lean and obese humans and in pig obesity models [75,76]. Supporting the similarity of pigs to humans, Furet et al. conducted a comparative analysis of human microbiota across various farm animals, including horses, cows, goats, sheep, rabbits, and pigs, using real-time polymerase chain reaction (PCR). This study found that while humans and all other farm animals could be distinguished from one another, pigs and rabbits had more compositionally similar fecal microbiota to humans in terms of metabolically active intestinal bacteria [75]. Notably, apart from rodents, pigs appear to be the only other host species stably colonized with human microbiota [77]. Unlike “humanized” mice [78], pigs born via cesarean section (i.e., not colonized by bacteria at birth) and later colonized with human GM develop relatively normal gastrointestinal morphology without evident immune system development deficiencies [79]. Remarkably, pigs colonized with human GM at birth develop the same or a higher number of IgA- and IgG-producing cells, CD4+ helper T cells (Th), and MHC class II antigen-presenting cells in the small and large intestines compared to control pigs experimentally colonized with porcine GM in a similar manner [80].

From the MIA perspective, although some porcine host defense polypeptides are specific and α-defensins are missing [81], most immune system proteins share structural and functional similarities with their human counterparts. In fact, the porcine IS is more similar to humans in >80% of the accounted parameters, whereas mice were more similar to humans in <10% [82]. The generation of porcine bone marrow-derived macrophages provides a model system for studying macrophage functional genomics that more closely resembles human biology than traditional mouse models [83]. Twitchell et al. used a gnotobiotic (Gn) porcine (Yorkshire crossbred) model of enteric dysbiosis to investigate the poor efficacy of the rotavirus vaccine in neonates from low- and middle-income countries (Twitchell et al. 2016). They colonized Gn pigs with human GM (of healthy and unhealthy neonates) and assessed the GM influence on vaccine immunogenicity and the microbiota’s response to human rotavirus (HRV) exposure. Their findings demonstrated that the compromised enteric immunity observed in human neonates could be replicated in the Gn pig model with an unhealthy human GM, suggesting potential future applications of this model for studying the microbiota’s role in various pathological stages related to the gut-immune axis [83].

Despite the advantages of this model, such as large litters, the possibility of standardizing housing conditions, high genomic and proteomic sequence homologies with human counterparts, and similar physiology to humans as omnivores, there are notable limitations associated with using pigs in microbiota studies [84,85]. The primary challenges associated with pigs are related to their size and the expense of housing and feeding them which complicate the acquisition of large sample sizes due to spatial and budgetary constraints [68,69]. Additionally, while genetic manipulation is possible, generating knockout and transgenic pigs is significantly more complex compared to rodents [84].

Other Mammals

Regarding the use of other large mammals in the MIA study, models such as Non-Human Primates (NHPs) and dogs (*Canis familiaris*) have been investigated.

For the dog model, the gastrointestinal tract is more similar in size and structure to the human GI tract compared to that of rodents discussed earlier. This similarity is relevant for translational research, as privately owned dogs are often exposed to the same environmental influences as humans [86]. The canine, human and mouse fecal microbiota show a high degree of metabolic and phylogenetic similarity [87]. Additionally, dogs (and cats) develop chronic inflammatory conditions that closely resemble human IBD [88]. These animals have been used as models to study the associations between specific compositional changes and the incidence of idiopathic canine IBD in different studies [87,89]. Regarding the IS responses, the canine model has been shown to be very similar to humans in terms of the proportion of T cells (CD8+ and CD4+) and B cells in the blood [90,91], the concentration of immunoglobulins [92], and the expression of T cell co-stimulatory molecules [93]. However, some unusual characteristics have been observed, such as the expression of CD4 by canine neutrophils, which warrants further investigation [94]. 

On the other hand, NHPs exhibit high similarity to humans in terms of anatomical structure, physiological metabolism, and immune system; however, few studies have systematically compared intestinal commensal microbiomes between humans and NHPs [95]. To date, studies have highlighted how primates (including monkeys) display immune responses very similar to those of humans, making them excellent models for vaccine and immunomodulatory therapy research [96]. Furthermore, a comparative study by Nagpal et al. GM microbiota appears to be more similar to that of NHPs compared to mice and rats [16]. In this scenario, Li et al. in 2020 demonstrated that short-term antibiotic-treated rhesus macaques can be used to study the host-microbiome niche of the intestinal mucosa and immune balance [97].

Despite the positive aspects that make the aforementioned models useful for research, both approaches have notable drawbacks, such as size and costs [98] Additionally, they present an even more pronounced issue regarding ethical consent for their use [99].

Zebrafish (*Danio rerio*)

The zebrafish (*Danio rerio*) is a freshwater omnivore from the carp family, native to the aquatic environments of India, Myanmar, Bangladesh, and Nepal [100]. Its use in research spans various fields, including embryology, tissue regeneration, molecular genetics, reproductive biology, and toxicology, thanks to several key advantages. These include its high reproductive rate, short lifespan, fully sequenced genome, transparent embryos and larvae and suitability for high-throughput in vivo screenings [101,102,103].

Mostly valuable for GM characterization, the zebrafish model is favored due to its genetic similarity to humans [104], structural and functional parallels between its intestine and those of mammals [105], optical transparency that enables advanced imaging techniques [106], and the ability to separately study the innate and adaptive immune systems [107,108]. These characteristics collectively make zebrafish an essential tool for exploring the complex interactions between the microbiota and host health. The GM role in the zebrafish immune system is pivotal for its development and function [109,110]. For example, Bates and colleagues highlighted that the microbiota helps regulate the number of neutrophils by modulating their production and activity through specific receptors and genes such as Myd88 and alkaline phosphatase [111]. Additionally, serum amyloid A (SAA), produced and secreted by intestinal epithelial cells, guides the behavior and migration of neutrophils, decreasing inflammation and defending against bacterial infections [112]. Brugman et al. further highlighted the critical IS role in managing the microbiota, showing that in wild zebrafish, the adaptive immune system controls Vibrio species overgrowth and that T lymphocytes are essential for maintaining microbiota balance and preventing bacterial overgrowth [113]. 

In addition, recent studies have explored the SCFAs’ impact on intestinal inflammation with promising outcomes. Cholan et al. discovered that zebrafish intestinal bacteria produce SCFAs, and that butyrate reduces inflammation through the hydroxycarboxylic acid receptor (hcar1) [114]. Morales Fénero et al. demonstrated that a mix of acetate, propionate, and butyrate alleviates intestinal inflammation caused by 2,4,6-trinitrobenzene sulfonic acid (TNBS) in zebrafish larvae. This treatment improved survival rates, preserved intestinal function and decreased inflammatory cytokines and neutrophil recruitment, although it did not prevent tissue damage or restore goblet cells. Despite TNBS-induced dysbiosis, SCFAs maintained normal bacterial levels. These findings indicate that zebrafish could serve as a valued model for studying SCFAs’ therapeutic potential in treating intestinal inflammation [115].

### 2.2. Invertebrates

*Galleria mellonella*

The invertebrate model of *Galleria mellonella*, or greater wax moth, is increasingly used as an alternative to rodent models for studies on bacterial and fungal virulence, viral infections, toxins and antimicrobial drugs due to the absence of ethical constraints, short life cycle, ease of handling and simple laboratory requirements [116,117,118]. Its immune system is structurally and functionally like that of mammals, making it a useful model for studying innate immune responses [116,119,120,121]. The cellular response involves hemocytes, the immune cells present in the hemolymph (equivalent to blood in vertebrates), responsible for processes such as phagocytosis, nodulation, and encapsulation [122,123,124]. As for the humoral response, it includes the release of antimicrobial peptides (AMPs) [125] and reactive oxygen and nitrogen species (ROS and RNS) [126,127]. Moreover, *Galleria mellonella* larvae develop a form of immune memory that enhances responses to repeated exposures to the same pathogen, although it is not comparable to the mammals’ adaptive immunity [128]. For instance, repeated infections with *Pseudomonas entomophila* improve immune responses, including increased hemolymph defensive activity, AMP presence, and expression of immunity-related genes, though this increased resistance is specific to *P. entomophila* and no other pathogens [129]. 

Recent studies highlight that *Galleria mellonella* cationic protein 8 (GmCP8), a recently identified AMP, shows antibacterial action against various pathogens and antifungal action against *Candida albicans*. This peptide damages the cell membranes of pathogens and inhibits specific proteases. Furthermore, the direct GmCP8 injection improves the survival of infected larvae, highlighting its role in humoral immunity [130]. Additionally, research by Gallorini et al. introduces a novel approach for immunophenotyping hemocytes from infected *Galleria mellonella* larvae. By detecting cell membrane markers typically expressed by human immune cells during inflammation and infection—such as CD14, CD44, CD80, CD163, and CD200—this study demonstrated significant analogies between *Galleria mellonella* larvae and humans. 

This advancement provides a valuable tool for pre-clinical evaluations of antimicrobial compounds, facilitating drug discovery and supporting clinical trials [131]. Studies on the *Galleria mellonella* microbiota reveal that an intact GM, dominated by *Enterococcus*, increases resistance to oral infections through the activation of AMPs and other immune responses, underscoring its role in strengthening defenses against specific pathogens [132]. Additionally, feeding larvae with polyethylene, polystyrene and beeswax shows significant changes in the microbiota, with an increase in *Pseudomonas* strains [133]. This promising result requires further investigation into potential multi-kingdom synergies in the plastic biodegradation process.

*Caenorhabditis elegans*

*Caenorhabditis elegans* (*C. elegans*) is a small nematode widely used in laboratory studies of embryonic development and cell differentiation due to its transparency. This organism has a constant number of somatic cells and a nervous system composed of only 302 neurons, allowing for the study of complex behaviors. Sharing many genes with humans, it is a valuable model for genetic research which began in 1962, thanks to Sydney Brenner, who received the Nobel Prize in 2002 for his work. 

This nematode was the first multicellular organism to have its genome sequenced in 1998, highlighting its fundamental role in developmental biology, genetics, and neurobiology. In addition to its well-established roles, *C. elegans* is a prominent model for studying immunity and host-microbiota interactions [134]. It possesses an innate immune system with evolutionarily conserved signaling mechanisms, such as the DAF-2 receptors and TLR (TOL-1) [135,136]. Since it lacks adaptive immunity and mobile immune cells, intestinal epithelial cells play a crucial role in defending it against ingested pathogens [137]. 

The *C. elegans* microbiota is predominantly composed of *Gammaproteobacteria* and *Bacteroidetes* [138]. Research by Singh and Luallen has analyzed factors influencing interactions between *C. elegans* and its microbiome, providing insights relevant to human biology [139]. For instance, interactions between *C. elegans*, the protective symbiont *Pseudomonas lurida MYb11*, and the pathogen *Bacillus thuringiensis Bt679* have shown that the nematode’s immune competence significantly affects its fitness under pathogen stress [140]. The protective action of these interactions can be evaluated through proteomic analysis, highlighting various pathways involved [141]. 

In addition, recent studies have highlighted the *C. elegans* utility for researching the effects of probiotics on obesity and diabetes. Various bacterial strains, such as Lactobacilli, exhibit probiotic properties in *C. elegans* models [142]. For instance, *Lactococcus lactis* subsp. *Lactis* improves locomotion and reduces lipid accumulation without significantly increasing longevity [143]. Similarly, *Lactobacillus cremoris* subsp. *Cremoris* has beneficial effects by stimulating the *SKN-1/Nrf2* signaling pathway [144]. In summary, *C. elegans* serves as a valuable model for probiotic research, providing crucial insights into the mechanisms through which strains like *Lactococcus lactis* and *Lactobacillus cremoris* enhance health. This model highlights significant molecular signaling pathways, such as *PMK-1/p38 MAPK* and *SKN-1/Nrf2*, which play essential roles in locomotion and lipid reduction. Overall, the versatile *C. elegans* applications in genetic, developmental and immunological studies underscore its relevance in advancing our understanding of microbiota-immune interactions.

*Drosophila melanogaster*

*Drosophila melanogaster*, commonly known as the fruit fly, is a valuable model organism in biological research due to its low maintenance costs, simple genetics, short generation times, and fully sequenced genome. It is especially useful for genetic and immunological studies. Similar to other organisms, *Drosophila melanogaster* possesses an innate immune system that is divided into humoral and cellular immunity [145]. The humoral immunity relies on the production of AMPs that protect the organism against microbial infections [146,147,148]. These AMPs, whose expression is regulated by NFκB-mediated immune signals, are made in specific tissues such as the fat body and released into the hemolymph [145]. Positively charged AMPs interact with negatively charged microbial membranes, destabilizing them and causing pathogen death. This process, known as the systemic immune response, is crucial for contrasting infections [149]. A notable discovery is the identification of a new antibacterial peptide with an O-glycosylated substitution. This post-translational modification enhances the peptide’s antibacterial activity compared to its natural form, highlighting the relevance of glycosylation for the peptide’s full biological effectiveness. Additionally, this defense system is highly regulated and can be activated in response to danger signals such as bacterial invasion, documenting the dynamic nature of *Drosophila*’s IS and its adaptability to microbe-rich environments [150]. On the other hand, cellular immunity involves hemocytes, which are abundant and diverse in Drosophila larvae [151]. These include plasmatocytes, crystal cells, and lamellocytes [151,152,153,154,155]. 

Immune signals activated in *Drosophila melanogaster* trigger the expression of immune response genes that are central to innate immunity. These signals start with the recognition of pathogen-associated molecular patterns (PAMPs) by host cell receptors, starting signal transduction mediated by adaptor proteins. The main immune signaling pathways in *Drosophila* are NFκB/Toll, NFκB/Imd, JAK/STAT and JNK [154]. The NFκB/Toll and NFκB/Imd pathways activated, respectively, by Gram-positive bacteria and fungi, and Gram-negative bacteria, lead to the synthesis of antimicrobial peptides [156]. The JAK/STAT pathway, activated by the association of Unpaired (Upd 1, 2, or 3) ligands with the Domeless (Dome) receptor, involves the phosphorylation of STAT, which regulates the expression of genes related to homeostatic and developmental processes [157]. Finally, the JNK pathway, induced by stress factors, activates the transcription factors AP-1 and FOXO, stimulating the production of antimicrobial proteins and involving responses to oxidative stress, homeostasis, embryogenesis, and apoptosis [158]. 

*Drosophila melanogaster* is also an excellent model for studying GM due to its simplicity. The microbiota is dominated by *Proteobacteria* and *Firmicutes* phyla and varies with the fly’s age and development [159]. Microbiota transmission occurs primarily through contaminated eggshells, which larvae ingest upon hatching, allowing microorganisms to colonize the larval gut and increase in diversity and abundance as the larvae develop. The resident microbiota provides numerous benefits, including strengthening intestinal integrity, shaping the epithelium, energy harvesting, protection against pathogens, and regulating host immunity [160,161,162,163,164]. The interaction between GM and IS during infections is more intriguing, suggesting complex relationships between pathogens and microbiota-dependent immune responses [165]. Studies on *Drosophila* have shown that the GM improves host survival in the presence of bacterial pathogens such as *Pseudomonas aeruginosa* and *Serratia marcescens* [166,167,168], by stimulating local production of antimicrobial compounds like ROS and AMPs and enhancing the intestinal epithelium’s ability to regenerate during and after infection [169]. Further investigations have revealed that the *Drosophila* GM can modulate the JAK/STAT, JNK and Imd signaling pathways, which are crucial for intestinal stem cell homeostasis and local immune responses [170]. Additionally, some GM species can transform bile acids and dietary substances into metabolites that influence the intestinal environment and immune balance, inducing basal immune responses through ROS [171,172,173,174,175,176]. 

To summarize, *Drosophila melanogaster* is an excellent model for studying the microbiota-immunity axis. Its simplicity, combined with the wealth of available genetic and immunological data, allows for a detailed exploration of the mechanisms through which the microbiota contributes to host health and immune defense. These studies not only enhance our understanding of microbiota-immunity dynamics but also suggest new therapeutic strategies for combating infections and other microbiota-related diseases.

**Table 1:** Overview of animal models for studying gut microbiome-immunity interactions: Comparative analysis of advantages, disadvantages, and research applications.

Animal Model	Advantages	Disadvantages	Applications
Rats	Closer physiology to humans; larger size allows complex studies; available disease models; easy maintenance; great overlap with the human microbiome.	Different diet/living conditions from humans; less microbiota variability.	Gut dysbiosis [27,28,29,30,31]; dietary/pharmaceutical effects [27]; microbiota analysis across GI tract [33]; age-related changes [34]
Mice (*M. musculus*)	Anatomical/physiological similarity to humans; similar bacterial composition at the phylum level.	Dietary and anatomical differences affect microbiota; some unique bacteria (e.g., SFB, Deferribacteres) not found in humans.	Diet-pathogens interactions and genotype effects on GM [35]; comparative microbial analysis [15,48]; immune system maturation [45,46,47]
Guinea pigs (*Cavia porcellus*)	Intestinal E-cadherin similarity; comparable microbial phyla and immune responses to humans.	Limited research; genus-level microbiota differences.	Human diseases models [53]; microbiota-immune axis exploration [53]
Rabbit (*Oryctolagus cuniculus*)	Immune system similar to human; intermediate size; well-characterized gut microbiota.	Higher costs and maintenance.	Infectious diseases (e.g., syphilis, TB) [60]; immune system research [60,62,63]; GM studies across life stages [64,65,66]
Pigs	Similar size and GI structure to humans; well-characterized microbiota; stable human microbiota colonization; gnotobiotic models.	Size and cost; complex genetic manipulation.	GI physiology and immune ontogeny [70,71]; diet-induced obesity; human microbiota colonization and immune responses [75,76,77,79,80]; vaccine efficacy and enteric immunity [83]
Non-Human Primates	High physiological and immune similarity to humans; similar gut microbiota.	High cost and size; ethical considerations.	Immune responses studies [96]; host-microbiome interactions [97]
Dogs (*Canis familiaris*)	Similar GI structure and immune responses to humans; comparable chronic inflammatory conditions (e.g., IBD).	Unique immune traits; high cost; ethical concerns.	GM and immune responses research [87,89]; comparative immune system studies [90,91,92,93].
Zebrafish (*Danio rerio*)	Genetic and structural parallels to humans; optical transparency; separate innate/adaptive immune systems.	Limited immune complexity; challenges in clinical translation.	Microbiota in immune system development [109,110,111,113]; SCFA and intestinal inflammation [114,115].
*Galleria mellonella*	No ethical constraints; short life cycle; similar immune system to mammals	Differences in the adaptive immunity compared to mammals.	Pathogens virulence [116,125,126,127]; immune memory [129]; antimicrobial studies [131]; microbiota research [132,133].
*Caenorhabditis elegans* (*C. elegans*)	Transparency for studies; simple nervous system; genetically tractable; conserved innate immunity.	Lack of adaptive immunity and mobile immune cells.	Microbiota-immunity interactions [134,139,140,141]; probiotic research [142,143,144].
*Drosophila melanogaster*	Low maintenance; simple genetics; well-documented immune pathways; simple microbiota dominated by Proteobacteria and Firmicutes.	Lacks of adaptive immunity.	Pathogens immune response [149,156,157,158]; GM and immune signalling studies [166,167,168,170,171,172,173,174,175,176].

SFB = segmented filamentous bacteria; GM = Gut microbiome; GI = Gastrointestinal; TB = Tubercolosis; IBD = intestinal bowel disease.

## 3. In Vitro Models

### 3.1. 2D Models

The animal models discussed previously are invaluable for expanding the understanding of the complex relationship between GM and IS. Specifically, GF and Gnotobiotic (GN) rodents are the most commonly used models to study MIA under both physiological and pathological conditions, and to establish causal links between specific bacteria and changes in immune responses [177]. However, these models have limitations, making translating data from animal systems to humans challenging. Besides ethical considerations, high costs and the time-consuming and labor-intensive nature of animal studies; in detail, animals often fail to accurately represent human conditions due to interspecies differences in gut topology and immune system, as well as differences in microbiome profile and molecular mechanisms involved in disease onset and progression [178].

For these reasons, in vitro models have been developed. Research in this area began with traditional cell cultures in culture plates. Cultivating a cellular monolayer to test with metabolites or bacterial extracts is easy to achieve, inexpensive, reproducible and provides raw results for preliminary studies. Indeed, there are numerous studies in which metabolites or bacterial extracts have been tested to study their interactions with human intestinal or immune cells [179,180]. The effects on the cells are then assessed through various tests, including permeability assays, reactive oxygen species (ROS) production, cell viability assays, and more [178].

In this regard, the most commonly used cell lines for studying the effects of the microbiota on intestinal cells are Caco-2, HT-29-MTX, T84, LS174T, and CCD 841 CoN. In a study by Lock et al., the Caco-2 and HT-29 lines were used to test the effect of conditioned media and microbial by-products from in vitro cultured microbiota on the human host, mimicking intestinal inflammation and cellular immunomodulation [181]. However, Pan et al. (2015) highlighted some disadvantages of using these lines. Caco-2 cells, originating from cancer cells, are homogeneous and do not produce significant amounts of mucin under normal growth conditions. HT-29 cells have a mucus layer, which is useful for microbiota research; however, they have similar drawbacks to Caco-2, as they are colon cancer-transformed cells and are not typically used to study barrier function due to their inability to form appropriate tight junctions. The T84 line, on the other hand, shares similar advantages and disadvantages but, unlike HT-29, T84 has been an excellent model for testing the effects of microbes and stress factors on epithelial barrier function due to its high transepithelial resistance (TER) properties [179].

The immune cell lines used to study immune responses to microbiota-produced metabolites mainly include macrophages (THP-1) and lymphocytes (Jurkat) [182]. Overall, when using these tumor cell lines, several factors must be considered, including culture conditions, the number of passages and whether they express the desired genes/proteins before determining if they are suitable for microbiological investigations [183].

Studying interactions with live bacteria in monolayer cultures poses challenges due to contamination risks, leading researchers to often use bacterial products instead. An advancement in this area is the development of co-culture systems, including Transwell co-culture systems and other devices that separate cells from live bacteria. These systems facilitate the study of interactions between live bacteria and host cells while decreasing contamination risks. For example, Magryś et al. employed a co-culture system of intestinal epithelial cells and macrophages to investigate how postbiotic fractions from *L. rhamnosus* and *L. plantarum* modulate immune responses to pro-inflammatory stimuli [184]. Zoumpopoulou et al. utilized a Transwell co-culture system with intestinal epithelial cells, dendritic cells, and various bacterial strains to study interactions while controlling contamination and experimental conditions [185].

Therefore, working with monolayer cells offers significant advantages, including high experimental reproducibility, continuous monitoring of culture conditions, accessibility, cost-effectiveness, and avoidance of ethical concerns. The general disadvantages of the above cell types include the lack of cellular diversity and the presence of a static environment far from the dynamics of the intestinal tract [186,187]. Another negative effect to consider, which is increasingly discussed, is the use of laboratory plastics, which have proven negative impacts on the environment, humans, and other organisms [188].

### 3.2. 3D Models

The 3D models currently used for studying the microbiota and its relationship with the immune system are spheroids and organoids. However, for several years, these two terms were often confused. For this reason, in 2012, the Intestinal Stem Cell Consortium established that a spheroid refers to a 3D culture that is only epithelial, whereas an organoid is a structure containing multiple cell types [189,190]. Unlike organoids, spheroids have much shorter lifespans in culture due to their irregular morphology, frequent disaggregation, and central hypoxia that leads to central necrosis [191]. So, spheroids are less commonly used in research in this field compared to organoids. Indeed, as previously mentioned, intestinal organoids are spherical culture systems derived from self-organizing pluripotent or adult stem cells that can differentiate into intestinal epithelial cells, producing structures akin to villi and crypts, thereby mimicking the architecture of the intestinal epithelium [186]. This model more faithfully replicates the intestinal architecture compared to two-dimensional systems. Moreover, since organoids can also be derived from patient biopsies, they are increasingly considered for personalized medicine applications [192]. To achieve three-dimensionality, various gels or scaffolds are used to support cell growth. These self-organized 3D tissue constructs exhibit an in vivo-like architecture, regional specification, and diverse cell subtypes, more closely mirroring the main characteristics of native human tissues than cell lines and animal models [193]. They are now widely used to study the microbiota in both physiology and pathology [194].

Regarding their use, several studies have aimed to create structures that simulate the human intestinal environment as closely as possible by combining intestinal organoids with IS. For instance, Dijkstra et al. developed a co-culture of tumor organoids and peripheral blood lymphocytes from patients with colorectal cancer [195]. In addition to the immune system, the microbiota can be introduced into the organoid. Microinjection has been a popular yet complex approach to achieve this goal in recent years [178]. Individual microbial species, pools of different microorganisms, or even fecal samples can be microinjected into the lumen of organoids, transforming them into miniaturized intestines [186]. Williamson et al. designed a microinjection platform using 3D-printed components. Their study injected a treated human fecal homogenate into the organoid, documenting that the microbial community persisted for up to four days [196]. Additionally, several protocols have been published to guide the use of this method [197].

However, this procedure requires specialized equipment, trained personnel, incurs high costs, and poses challenges for reproducibility [178]. Even setting up an organoid culture is non-trivial, as replicating the microstructures of the human intestine, such as villi, is complex [195]. Moreover, like organoid creation, microinjection lacks standardized guidelines, making it difficult to ensure data reproducibility and comparability. A significant drawback of organoid systems is the lack of inter-organ communication. Human organoid systems essentially mimic a part of the human body, not the entire body. Thus, they are limited to reproducing the microphysiology of a specific organ or tissue, a limitation that must be considered before delving into this exciting field. Nonetheless, efforts are already underway to overcome this limitation [198]. Recent advancements in tissue engineering have led to improved 3D tissue models that allow the integration of different cell types and better mimic the in vivo microenvironment. The advent of organoid research has further enhanced in vitro models, enabling better recapitulation of tissues’ complexity such as the intestinal epithelium. However, these models usually do not allow for mechanical signals such as fluid flow and peristalsis-like mechanical deformations [199].

### 3.3. Microfluidic/On-Chip Models

One of the most promising applications of organoids to date involves culturing them under perfusion in microfluidic devices [200,201]. Recent progress in bioengineering, millifluidics and microfluidics has led to the development of increasingly sophisticated devices that approximate the complexity of the human intestine [186]. Microfluidics is defined as the technology of systems that process or manipulate small amounts of fluids [202]. By 2010, nearly 10,000 articles had been published on this technology, but it has more recently been applied to biological studies in vitro [203]. It offers numerous advantages, such as the use of very small quantities of samples and reagents, high resolution and sensitivity, and compact analytical device sizes [204]. As a result, lab-on-a-chip platforms and organs-on-chips have become crucial technologies for studying biological processes. They enable fine control over cellular environments, including perfusion, nutrient supply, waste removal, and the maintenance of pH and oxygen gradients [200].

Today, various models on the market can mimic the complex interactions occurring in our bodies due to the connection between different tissues. For instance, Beaurivage et al. developed a model for IBD evaluation, using a commercial platform and demonstrating the complexity and versatility of these technologies [205]. Kim et al. documented that a microfluidic gut-on-chip technology, which exposed cultured cells to physiological movements similar to peristalsis and fluid flow, could induce human Caco-2 cells to undergo spontaneous morphogenesis and produce three-dimensional villus-like intestinal structures [206]. Additionally, Kasendra et al. recently developed a small intestine-on-chip device using biopsy-derived organoids [207].

To include the microbiota in gut-on-chip systems, different approaches have been used. Most studies have employed single strains of pathogens [208], probiotics [209] or combinations of both [210]. Co-cultivation of human and microbial cells under aerobic conditions is the most utilized practice. However, to more accurately mimic the human intestinal environment, several platforms have been developed to include an anaerobic compartment with selected strict anaerobes or a more diverse microbiota derived from fecal samples [211]. For instance, Jalili-Firoozinezhad and coll. used a microfluidic gut-on-chip to co-culture intestinal epithelium with stable communities of both human aerobic and anaerobic microbiota. This system allows for real-time control and assessment of physiologically relevant oxygen gradients. By establishing a trans-luminal hypoxia gradient, this chip improved intestinal barrier function and enabled a more representative microbial diversity compared to aerobic co-culture conditions [212].

In another development, Marzorati et al. created the Host-Microbiota Interaction (HMI) module, a microfluidic system designed to study the response of a monolayer of Caco-2 cells to metabolic products from bacterial biofilms. This module features two flow chambers separated by a semipermeable membrane, with bacteria introduced into the upper chamber [213]. Additionally, gut-on-chip models have been applied to study non-bacterial components of the microbiota, such as viruses, parasites, and phages, as well as live biotherapeutic products (LBPs) and microbial toxins [214].

The IS integration into these models has already begun and continues to be studied and implemented. There are already several lab-on-chip models that incorporate intestinal and immune cells, especially for studying IBD [180,215]. Generally, these models are quite complex and usually rely on the most common stacked microfluidic channels separated by a porous membrane [199].

Despite their advantages and potential, these models show some limitations. Many lab-on-chip systems face challenges related to productivity, limited readout techniques, and the need for specialized equipment. Although they are less expensive than in vivo studies, costs can still be significant [199]. Another issue is the variability in parameter values between individuals and different regions of the intestinal tract, which complicates the identification of key factors influencing GM composition [216]. Additionally, microfluidic and lab-on-chip technologies face technical hurdles, such as accurately replicating MIA and modeling variations in microbiome composition along the mucosal-luminal axis. Nevertheless, with ongoing advancements and optimizations, these models have the potential to greatly enhance scientific research and deepen our understanding of gut, microbiota, and immune system interactions [217].

## 4. In Silico Models

The advent of computational technologies has significantly transformed research, enabling the use of in silico models to explore the intricate MIA interactions. These models are pivotal in investigating the dynamic relationships between GM and host biology, enhancing our comprehension of MIA and the influence of gut microbes on host’s biology [218]. 

The main goals of in silico models include:

1. Simulation of Microbial Ecosystems: In-silico models allow scientists to simulate the complex GM ecosystem. By using computational techniques, researchers can model the growth, interaction, and metabolic processes of diverse microbial communities, providing insights that are challenging to obtain through traditional experimental methods [219,220].
2. Host-microbe interaction: these models simplify the study of host-microbe interactions at multiple levels, from molecular to systemic. They can integrate multi-omics data to predict how microbial metabolites affect host cells and vice versa [221,222].
3. Immune system modulation: understanding how GM influences the host’s IS is crucial for developing therapeutic strategies. In-silico models can simulate immune responses to various stimuli, helping to identify potential targets for immunomodulation [223].
4. Disease modeling: computational models are essential for exploring how disruptions in the GM contribute to inflammatory and autoimmune diseases [224]. They enable the identification of microbial signatures associated with these diseases and predict the effects of potential treatments.

**Figure 1:**
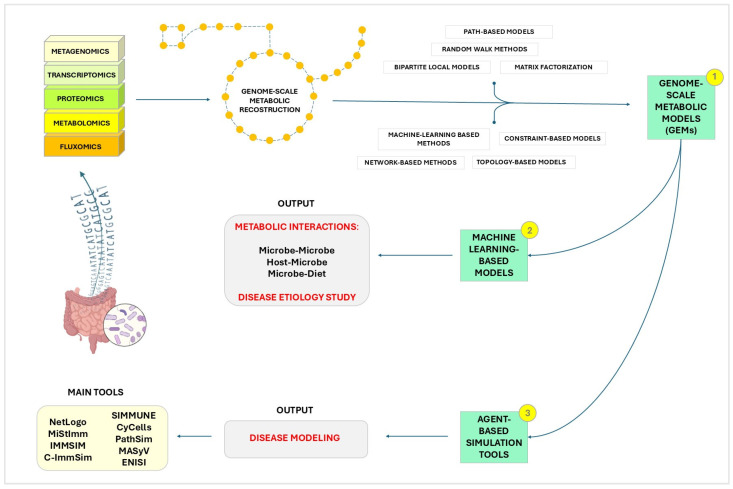
Schematic overview of the main in-silico models and tools to study metabolic interactions, disease etiology and disease modeling. The figure summarizes the three prominent types of in-silico models used to evaluate the MIA that will be discussed in detail in the next paragraphs: (1) multi-species ecosystem models/Genome scale metabolic models (GEMs), (2) machine learning-based models and (3) agent-based simulation (ABS) tools.

In the next sections, we will explore three prominent types of in silico models used to study the MIA: multi-species ecosystem models, machine learning-based models and agent-based simulation tools (Figure 1). Each of these approaches brings unique strengths and capabilities to the table, offering diverse perspectives and methodologies for advancing our understanding of the complex interactions within the GM and its impact on immune function.

### 4.1. Multi-Species Ecosystem Models

Traditionally, microbiome research has focused on identifying and quantifying the microbial communities present in the gut, focusing on taxonomy but often neglecting the intricate network of interactions among these microbes and their effects on the environment, namely the host. To gain a thorough, system-level understanding of the microbiome and its communication with the host IS, it is crucial to consider these interactions [225]. Computational multi-omics approaches have been instrumental in this regard [226,227], allowing for the analysis of the diverse and dynamic GM populations. In fact, the data integration from genomics, proteomics and metabolomics, has led to the development of in silico models that enable the evaluation of host-microbe interactions at various levels, from molecular to systemic, predicting the effects of microbial metabolites on host cells and vice versa in both physiological and pathological conditions [228,229]. 

Indeed, the need to employ systems biology to investigate the microbiome has driven the development and analysis of in silico system-level metabolic models [222,230]. We know that microbiome metabolism is a complex composite of the metabolic activity of billions of microbial cells from various species. By treating the entire microbiome as a single supra-organism [231], in silico models can be constructed directly from metagenomic data. This approach is particularly suitable when (i) many gut-dwelling species are difficult to isolate and sequence, making community-level models essential; (ii) for studying the overall activity of the microbiome and its interactions with the host [232], considering the overall metabolism of this network as a co-metabolism. These models can, for instance, be used to explore the potential exchange of metabolites between the microbial community and the gut environment. Furthermore, integrating these models with human metabolic models enables the examination of metabolic dependencies between GM and host, similar to the study of interactions between a single microbial endosymbiont and its host [232]. 

For instance, Genome-scale metabolic modeling (GSMM) is a modeling approach that has been used to study microbial metabolism in the human gut and the interactions between microbes and host [221,233,234,235]. Genome-scale metabolic models (GEMs) of human microbes provide a robust framework that integrates multiple omics datasets such as transcriptomic, proteomic, metagenomic, metabolomics and fluxomics (a branch of systems biology that analyzes the metabolic fluxes within a biological system, namely the rate at which metabolites are produced, consumed and transformed within a community of microbes), providing a comprehensive system biology platform to analyze and infer diet-microbiome, microbe-microbe and host-microbiome interplays under physiological conditions and to build condition-specific personalized community models of the gut microbiome [236] to study the effect of microbial changes on disease. For instance, the integration of metagenomic data of patients with Crohn’s disease with genome-scale metabolic models has allowed the construction of personalized in silico microbiotas for the prediction of SCFA levels as a consequence of different dietary treatments [236]. 

By combining multiple single-species models that provide insights into the metabolic functions of individual species, such as topology and constraint-based models [230,237], the human microbiome can be studied as an ecosystem community model. Using various computational approaches, such as reverse ecology, it is possible to predict the type and extent of interactions within the community and its environment [231]. This includes inferring whether a bacterial species can influence the metabolism of host tissue cells, thereby enhancing our understanding of the microbiome-host metabolic interplay. 

**Figure 2:**
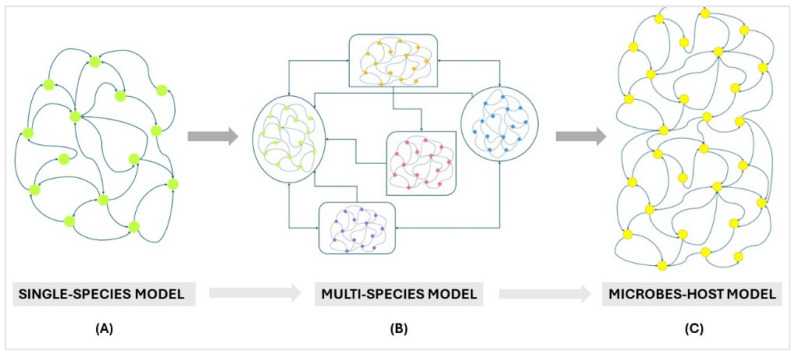
Integration of single-species models with host metabolism models to study interactions between the gut microbiota (GM) and the host through a comprehensive supra-organism model. (**A**) Each colored network represents the metabolic model of a single microbial species. (**B**) By combining multiple single-species models, a multi-species model can be constructed, simulating functional exchanges between different microbial species within a community. (**C**) Integrating this multi-species model with host biological data creates a microbe-host model, enabling the study of interactions between microbes and the host.

According to Garjan et al., there are six primary computational methods used to predict microbial disease: path-based methods [238], random walk methods [239,240], bipartite local models [241], matrix factorization [242,243], machine learning-based methods [244] and network-based methods [245,246]. These techniques aim to predict links between microbes and diseases. However, topology and constraint-based methods can complement these approaches by offering insights into the structural organization and functional limitations within microbial communities. Though being mainly used to model single organisms or cells, it is possible to integrate them with metagenomic data to study the microbiome and its interaction with the host on a system level (Figure 2). One such integration has led to the development of the Microbiome Modeling Toolbox [247], through which we can determine pairwise metabolic interactions regarding metabolic exchanges between two metabolic reconstructions, such as microbe-microbe and host-microbe, and to build and simulate a personalized microbial community model in different conditions, e.g., under different diet regimens, using specific microbes and their relative abundance [248] within a sample. 

Lastly, by merging the predictive capabilities of machine learning-based methods with the structural understanding provided by topology [249] and constraint analysis [250] mentioned above, researchers can gain a more holistic view of how microbial communities impact disease. This integrated approach has led to the development of machine learning software that enhances our understanding of the complex interactions that exist between GM and health [251].

### 4.2. Machine Learning-Based Models

Machine learning-based models have emerged as powerful tools for MIA exploration, offering unique capabilities in handling large-scale datasets and uncovering complex patterns within them [252]. These models provide a promising approach to simulate and predict the intricate interactions between the GM and the host immune system [253]. 

The computational methodology for predicting the human-microbial interactions involves exploring the global landscape of potential inter-species protein interactions within different human microbiomes and assessing their impact on human cellular pathways [254]. This approach aims to estimate the influence of these interactions on the host and provides insights into the complex network of relationships between human and microbial proteins, which includes the interactions between viruses and human proteins. By using computational tools and protein interaction mapping techniques, researchers can predict and analyze these interactions, shedding light on the intricate dynamics of the GM-immunity axis [255].

Machine learning-based models stand out due to their ability to integrate various types of biological data, including genomics, proteomics and metabolomics, to generate comprehensive predictions about host-microbe interactions. These models are principally adept at identifying patterns and correlations that might be overlooked by traditional analytical methods. For instance, by employing computational predictions based on sequence similarities, gene-order conservation, and protein structural data, researchers can capture interactome networks and identify potential links between different diseases [256]. This integrative approach allows for the identification of shared molecular pathways and interactions with gut microbes that may underlie disease relationships, providing valuable insights into the etiology, treatment, and development of many microbe-related diseases.

One of the significant advantages of machine learning algorithms is their ability to handle the complexity and heterogeneity of microbial communities’ data. By processing large datasets, these models can discern subtle patterns and associations that inform our understanding of how specific microbial populations influence immune responses. For example, machine learning models can predict microbial disease associations, shedding light on how dysbiosis can impact the host IS and overall health.

Moreover, these software tools can incorporate network-based methods, offering insights into how microbiota can influence immune responses and vice versa [231]. Network-based approaches allow researchers to map out the interactions between different microbial species and the host immune system, highlighting key nodes and pathways that may be critical for maintaining immune homeostasis. These insights are crucial for unraveling the molecular commonalities between clinically related diseases, such as autoimmune diseases, even when they do not share disease genes.

Furthermore, machine learning models can integrate topology and constraint-based methods, enhancing our understanding of how microbial communities impact immune responses and overall health [257]. Topology-based approaches focus on the structural properties of biological networks [258], identifying critical connections and hubs that play essential roles in mediating host-microbe interactions. Constraint-based models, on the other hand, use mathematical frameworks to simulate metabolic networks, predicting how changes in microbial composition can influence metabolic outputs and, consequently, immune function [250,259].

In addition to predictive modeling, machine learning approaches are useful for hypothesis generation and experimental design. By analyzing large datasets, these models can generate new hypotheses about potential mechanisms of MIA interactions, managing experimental studies [260,261]. For instance, machine learning algorithms can identify candidate microbial metabolites that might modulate immune responses [262,263], providing targets for experimental validation and therapeutic development [264,265].

Another area where machine learning-based models excel is in the personalization of medicine. By integrating patient-specific data, these models can predict individual responses to targeted therapies for microbiota-related diseases such as IBD [266], enabling the development of personalized treatment strategies. This is particularly relevant in the context of diseases like IBD and other autoimmune disorders [266], where GM plays a significant role in disease progression and response to treatment [267].

In addition, the integration of machine learning with high-throughput sequencing technologies has facilitated the discovery of novel microbial species and functional genes that may be critical for immune modulation [268,269]. These discoveries expand our understanding of GM diversity and functional capacity, providing new avenues for therapeutic intervention.

Overall, the application of machine learning-based models in studying the GM-immunity axis represents a significant advancement in our ability to understand and manipulate this complex system. By leveraging the power of computational tools, researchers can gain deeper insights into the interactions between the GM and the immune system, paving the way for novel therapeutic strategies and improved disease management.

### 4.3. Agent-Based Simulation Tools

Over recent decades, different mathematical and computational models have been developed to simulate and describe the processes and characteristics of the immune system. These models can generally be categorized into two broad classes based on their modeling approach: top-down and bottom-up approaches [270]. The top-down approach focuses on estimating the average behavior at a macroscopic level, thereby modeling entire populations rather than individual entities. This approach allows for the representation of a large number of entities. The most traditional and widely recognized top-down models involve ordinary and partial differential equations, including stochastic differential equations which incorporate random components to reflect individual variability or environmental fluctuations caused by statistical noise [271]. Nevertheless, these models overlook individual interactions, though having the advantage of being grounded in well-established mathematical theory, facilitating, in some cases, analytical studies and asymptotic analysis, i.e., the understanding of the behavior of algorithms as their input increases [272,273]. However, for complex biological scenarios, these models can become unwieldy, necessitating approximations. 

On the other hand, the bottom-up approach operates at a microscopic level, where individual entities (agents) and their interactions are explicitly modeled, with the system’s overall behavior emerging from the collective local interactions [270]. This approach allows for more precise modeling of localized immunological processes, reducing the need for the broad assumptions typical of top-down models. In this context, agent-based simulation (ABS) tools are the most commonly used bottom-up methods in immunology studies [274].

While machine learning (ML) techniques have proven invaluable in analyzing large datasets and predicting patterns within the microbiome [275], ABS tools offer a complementary approach by enabling detailed modeling of interactions among individual microbial agents and their environment [276].

ABS is a computational methodology that focuses on the behaviors and interactions of autonomous agents to explore the emergent properties of complex systems [277]. In the context of GM research, each agent can represent an individual microorganism or a group of microorganisms, each with distinct characteristics and behavioral rules [278]. These agents interact with each other and their environment, allowing researchers to simulate and analyze how various factors—such as diet, antibiotics and host immune responses—affect the overall dynamics of the microbial community.

The primary advantage of ABS tools lies in their ability to model the non-linear interactions and stochastic events that drive the behavior of the microbiota [279]. Unlike traditional modeling techniques, which often rely on averaged parameters and assumptions of homogeneity, ABS captures the diversity and individuality of microbial entities [280]. This approach provides a more nuanced understanding of how specific interactions contribute to the stability, resilience, and functionality of the gut microbiome [281].

By using ABS tools, researchers can conduct virtual experiments to test hypotheses, explore “what-if” scenarios, and predict the outcomes of interventions [282]. These simulations can illuminate the underlying mechanisms of microbial interactions with the host IS and offer insights into the emergent behaviors of the microbiota as a whole [283]. These computational tools are essential, for instance, to explore how dysbiosis contributes to the immune responses that underlie diseases such as IBD, diabetes and even neurological disorders. They enable the identification of microbial signatures associated with these diseases and predict the effects of potential treatments.

There are various computational software tools and modeling techniques available to simulate immune responses and potential associations between microbes and disease. These tools allow researchers to model and analyze the interactions within the immune system accurately. Among these, NetLogo has been widely used to dive into the study of immunological dynamics. In fact, NetLogo stands out as a free and open-source programming language and integrated modeling suite that is quite easy to use for beginners due to the fact that it supports 2D or 3D drawn agents, as well as supplying many examples and how-tos [270]. This program can be used to model basic innate and adaptive immune responses and inflammation, but also to model autoimmune diseases such as Multiple Sclerosis [284].

Since such diseases rely on a dysregulation of immunity, which can be considered as a communicative system that maintains internal homeostasis by detecting and processing environmental signals, during the last years there has been a growing need to manage this complexity through complex computational tools that allow “dry-laboratory” experiments to be complementary to traditional in vivo/in vitro ones [285]. Several immune simulators have been developed to offer a programming framework capable of integrating existing immunological knowledge and modeling various aspects of immune dynamics. The Microscopic Stochastic Immune System Simulator (MiStImm) is one such tool that uses an agent-based modeling technique to simulate components of adaptive immunity, including T cells, B cells, antibodies, interleukins, danger signals, self-cells, foreign antigens and their interactions [286]. All of these major components, referred to as “agents”, represent the nodes of a dynamic immune network, where the links signify possible interactions between different elements. The immune network evolves over time, driven by random events in a step-by-step manner. In fact, the model simulated by MiStImm has a stochastic nature, which allows for random interactions, being useful for simulating key immune processes in which events happen randomly, like B cell affinity maturation and the selection of specific T cell clones [286]. Another tool, C-ImmSim, integrates the amino acid sequence of antigenic epitopes with lymphocyte receptors to simulate the immunological response [287]. C-ImmSim is the evolution ofIMMSIM, which enables the implementation and simulation of detailed immune response models [288]. With C-ImmSim many features not included in the previous version allow the study of humoral and cellular immunity as well as chemical mediators of cell communication such as cytokines. This computational tool can be used to study complex aspects of immunity that happen in a specific environment, such as thymus and bone marrow, while taking into account, compared to MiStImm, a wider range of cellular and molecular entities [287]. Furthermore, the Basic Immune Simulator (BIS) is an agent-based model that studies interactions between cells of the innate and adaptive immune systems in response to microbial infections [289]. Platforms like the ones mentioned above integrate others like SIMMUNE [290], Reactive Animation [291,292] and SIS (Synthetic Immune System) [293], which are designed to simulate immune reactions by allowing users to define interaction rules. The aim of these simulators is to make it easy to modify and explore different rules to study their effects on immune responses. While some simulators are more flexible than others, IMMSIM, for instance, enables users to adjust parameter values to alter specific aspects of immunity, like turning off the humoral responses to study the consequent outcome [294]. Reactive Animation offers even greater flexibility by allowing users to select between different theoretical models of uncertain interactions. Although the effectiveness of these platforms varies, some have contributed valuable insight to immunological research. For instance, IMMSIM has been used to study immune tolerance [295] and autoimmunity [287].

Another type of simulator, known as disease simulator, provides a versatile programming framework that can be adapted to model various diseases, being specifically designed to replicate different host-pathogen interactions and being generally more user-friendly and easier to calibrate compared to immune simulators. Users can fine-tune parameters such as cytokine binding rates and infection spread rates to simulate various diseases. Examples of such simulators include CyCells [296], PathSim [297] and the MASyV modules ma_immune and ma_virions [296].

These are just some examples of the main tools that provide a robust platform to explore the dynamics of immune responses in various contexts and scenarios. Overall, these computational software tools are instrumental in advancing our comprehension of immune responses in general by facilitating the simulation and analysis of complex immune system behaviors, interactions, and responses to different stimuli.

ABS tools have not only been used to study immunological disorders in general and responses to microbial stimuli deriving from bacterial or viral pathogens but also represent a valuable means to predict and study immunological consequences in response to stimuli related to the GI content, such as commensal microbes. In this regard, the ENteric Immunity Simulator (ENISI) is a simulator of the gastrointestinal immune mechanisms in response to resident commensal bacteria and/or invading pathogens and the effect on the development of intestinal lesions and immunopathologies [298,299]. This tool is developed to model the competing inflammatory and regulatory immune pathways in the gut as individual immune cells interact with both commensal and foreign bacteria. For instance, ENISI has been utilized to replicate a standard inflammatory response to foreign bacteria and the immunopathological consequences of autoimmunity against microbiota, involving 10^6^ cells [299]. ENISI represents a clear example of how such agent-based simulators are crucial to test the plausibility of in vitro experiments and in vitro/in silico observations, leading to the conduction of low-cost, preliminary experiments of proposed interventions, treatments [223] and potential immunological mechanisms not yet studied in vitro [299]. In fact, with ENISI and other ABS tools, immunologists can explore and better understand the complexities of enteric disease pathology. By simulating immune responses within an in silico gut environment, these tools allow researchers to test and develop hypotheses regarding the interactions between immune cells, commensal bacteria and pathogens. These simulation platforms are particularly valuable for investigating the dynamics of inflammatory and regulatory pathways [299], providing a controlled and customizable environment in which various scenarios can be modeled. Researchers can use such ABS tools to conduct preliminary computational experiments, refining their approaches before transitioning to more costly and time-consuming in vitro or in vivo studies. Moreover, ENISI’s capacity to model specific immune responses and disease outcomes [223] facilitates the identification of potential therapeutic interventions, offering insights that can inform experimental designs and drive forward more targeted and efficient investigations in the laboratory. By bridging the gap between computational simulations and experimental in-vivo/in-vitro research, ENISI serves as a versatile tool in advancing our understanding of gut immunity and disease. 

## 5. Conclusions

The exploration of diverse models for studying microbiota-immune system interactions reveals a nuanced landscape, with each model offering distinct advantages and limitations (Table 2). In vivo models provide a comprehensive biological context essential for understanding the intricate dynamics of the microbiome within an entire organism. However, they present significant ethical and practical challenges, such as high costs and inherent variability, which can complicate their use. Conversely, in vitro models, including two-dimensional and three-dimensional systems as well as advanced gut-on-chip models, offer the opportunity to reduce animal use while allowing for greater experimental control. Although these models are less expensive than in vivo studies, they still require specialized materials and advanced technological infrastructure. Nonetheless, in vitro models remain critical for mechanistic studies, enabling the isolation of specific variables and contributing to a deeper understanding of microbiota-immune system interactions.

In silico approaches emerge as well promising for the future of microbiome research. These computational models offer a sustainable, cost-effective solution that significantly reduces the environmental impact of experimental research. In silico methods can integrate data from in vivo and in vitro models to conduct large-scale simulations and generate accurate predictions, providing a powerful tool for understanding complex biological interactions. However, the success of in silico models depends on robust experimental validation and continuous optimization to ensure their accuracy and reliability.

Looking forward, the future of MIA research is likely to focus on the increasing importance of in silico models, given their sustainability and efficiency. The integration of in vivo, in vitro, and in silico methods promises to offer a more comprehensive and multidimensional perspective, but with a strategic emphasis on the in silico approach. This approach can accelerate scientific discoveries while promoting more ethical and environmentally sound research practices. Future studies should prioritize the development and refinement of in silico models, with the goal of harnessing their full potential to revolutionize our understanding of the role of the microbiome in health and disease.

## 6. Search Strategy from Repository

We conducted a comprehensive literature search focusing on publications that discuss in vivo, in vitro, and in silico models. Our aim was to identify studies that investigate the current and future approaches to understanding gut microbiome-immunity dynamics, particularly those that bridge experimental and computational models.

We utilized the PubMed database, leveraging the Medical Subject Headings (MeSH) tool to ensure a precise and targeted search. For our search, we selected the following key MeSH headings: “Gut Microbiota”, “Immune System”, “Models, Biological”, and “Computational Biology”. To enhance the focus of our search, we used boolean operators to combine these headings. The combinations included: (“Gut Microbiota” [Mesh]) AND “Immune System” [Mesh]; (“Gut Microbiota” [Mesh]) AND “Models, Biological” [Mesh]; (“Gut Microbiota” [Mesh]) AND “Computational Biology” [Mesh]; (“Immune System” [Mesh]) AND “Models, Biological” [Mesh]; (“Immune System” [Mesh]) AND “Computational Biology” [Mesh].

Throughout the selection process, we prioritized studies that offered comprehensive data, including Meta-Analyses and Systematic Reviews, as these sources consolidate existing knowledge and provide broader insights into the field. We also included recent Randomized Controlled Trials to capture current experimental approaches and Reviews that explore future implications and emerging trends in gut microbiome research. This search strategy ensured a thorough and well-rounded selection of publications, encompassing various study designs and methodologies, to provide a comprehensive overview of the state and future directions of research on gut microbiome-immunity.

## Figures and Tables

**Table 2:** Comparative Analysis of in Vivo, In Vitro, and In Silico Models: Advantages and Disadvantages in Studying Gut Microbiota-Immune System Interactions.

Models Used for Studying the Gut Microbiota-Immune System Interaction	Advantages	Disadvantages
In Vivo	Comprehensive biological context; manipulates microbial compositions; insights into dynamic interactions.	Ethical concerns; high cost; labor-intensive; variability; limited human applicability.
In Vitro	Ethical; high experimental control; cost-effective; detailed mechanistic studies.	Lacks full biological complexity; requires advanced technology; some models don’t replicate gut environment.
In Silico	Cost-effective; large-scale simulations; models complex interactions; aids experimental design.	Requires robust data for accuracy; potential oversimplification of biological processes.

## Data Availability

No new data were created or analyzed in this study. Data sharing is not applicable to this article.
